# Text Message-Based Intervention Targeting Alcohol Consumption Among University Students: Findings From a Formative Development Study

**DOI:** 10.2196/mhealth.5863

**Published:** 2016-10-20

**Authors:** Kristin Thomas, Catharina Linderoth, Marcus Bendtsen, Preben Bendtsen, Ulrika Müssener

**Affiliations:** ^1^ Division of Community Medicine Department of Medical and Health Sciences Linköping University Linköping Sweden; ^2^ Database and Information Techniques Department of Computer and Information Science Linköping University Linköping Sweden

**Keywords:** SMS intervention, university student drinking, formative research

## Abstract

**Background:**

Drinking of alcohol among university students is a global phenomenon; heavy episodic drinking is accepted despite several potential negative consequences. There is emerging evidence that short message service (SMS) text messaging interventions are effective to promote behavior change among students. However, it is still unclear how effectiveness can be optimized through intervention design or how user interest and adherence can be maximized.

**Objective:**

The objective of this study was to develop an SMS text message-based intervention targeting alcohol drinking among university students using formative research.

**Methods:**

A formative research design was used including an iterative revision process based on input from end users and experts. Data were collected via seven focus groups with students and a panel evaluation involving students (n=15) and experts (n=5). Student participants were recruited from five universities in Sweden. A semistructured interview guide was used in the focus groups and included questions on alcohol culture, message content, and intervention format. The panel evaluation asked participants to rate to what degree preliminary messages were understandable, usable, and had a good tone on a scale from 1 (very low degree) to 4 (very high degree). Participants could also write their own comments for each message. Qualitative data were analyzed using qualitative descriptive analysis. Quantitative data were analyzed using descriptive statistics. The SMS text messages and the intervention format were revised continuously in parallel with data collection. A behavior change technique (BCT) analysis was conducted on the final version of the program.

**Results:**

Overall, students were positive toward the SMS text message intervention. Messages that were neutral, motivated, clear, and tangible engaged students. Students expressed that they preferred short, concise messages and confirmed that a 6-week intervention was an appropriate duration. However, there was limited consensus regarding SMS text message frequency, personalization of messages, and timing. Overall, messages scored high on understanding (mean 3.86, SD 0.43), usability (mean 3.70, SD 0.61), and tone (mean 3.78, SD 0.53). Participants added comments to 67 of 70 messages, including suggestions for change in wording, order of messages, and feedback on why a message was unclear or needed major revision. Comments also included positive feedback that confirmed the value of the messages. Twenty-three BCTs aimed at addressing self-regulatory skills, for example, were identified in the final program.

**Conclusions:**

The formative research design was valuable and resulted in significant changes to the intervention. All the original SMS text messages were changed and new messages were added. Overall, the findings showed that students were positive toward receiving support through SMS text message and that neutral, motivated, clear, and tangible messages promoted engagement. However, limited consensus was found on the timing, frequency, and tailoring of messages.

## Introduction

An increasing proportion of the global burden of disease is due to alcohol consumption. In 2010, alcohol caused 5.5% of the total burden of disease and approximately 5 million deaths, which represents a 30% increase from 1990 [1]. Drinking of alcohol among university students is a global phenomenon and heavy drinking is a part of the social norm despite potential negative consequences [2,3]. Approximately half of all young adults in Sweden attend university making the health and well-being of this group an important public health concern [4]. Thus, it is important to develop cost-effective interventions that are able to reach large numbers of students.

There is emerging evidence that short message service (SMS) text messaging is a cost-effective method to support behavior change [5]. A review of controlled trials of SMS text message-based interventions showed that eight of nine studies found support for SMS text message-based interventions on behavior change. Outcomes included weight loss, smoking cessation, and diabetes management [6]. Another review of 14 studies identified evidence supporting SMS text message-based interventions on behavior change in all but one study. Aspects such as tailored content and interactivity were found to be important [7]. Furthermore, a 12-week SMS text message-based intervention targeting heavy drinking among young adults was found to have an effect on the number of heavy drinking days and the number of drinks per drinking day compared with baseline data [8].

Furthermore, SMS text message-based interventions have been shown to be highly accessible in that messages are likely to be read within minutes of being received and that receiving and reading messages require limited time and effort by the user [9-11]. SMS text message-based interventions can enable continuous, real-time, brief support in a real-world setting [9,12,13]. The real-time aspect may even serve a purpose beyond the actual content or meaning of messages [14]. SMS text message-based interventions have been designed based on existing evidence-based interventions and have adopted techniques similar to face-to-face treatments, such as tailored advice or goal setting [9,14]. Thus, SMS text message-based interventions could be cost-effective and applicable to many health behaviors [5,15,16]. However, it is still unclear how effectiveness can be optimized through, for example, message content and structure [17], or how user interest and adherence can be maximized [15]. User compatibility is seldom evaluated; at best, it is performed after delivery of the intervention [15].

Formative research methods, which consider the input from users and experts, have been successfully used previously [18]. The aim is to improve the design and performance of an intervention through input on content and regimen from the target group, experts, and prevalent theory. By engaging users in the development of the intervention, it is believed that compliance and fidelity to the intervention will increase; however, few studies merge the knowledge and interests of users and investigators [15]. Also, more studies describing the process of developing interventions in a transparent way are needed [19].

Furthermore, the use of theory has been limited in the development of SMS text message-based interventions [6,15,20] making it difficult to identify why and how an intervention results in desired outcomes. The association between using theory as a basis for designing interventions and effectiveness is not well understood [20]. It is unclear if it is more effective to use single or multiple theories in intervention development [17]. A multitheory approach without a clear rationale may not always be as effective as an intervention with systematic use of a single theory [20,21]. Taxonomies of behavior change techniques (BCTs) have been developed to aid the process of identifying effective features of an intervention [22,23].

This study reports on the findings from formative development of a text messaging alcohol intervention aimed at university students. The research builds on previous work from the AMADEUS research program [11,24,25].

## Methods

### Intervention Revision Process

The first version of the intervention, which included 57 messages, was generated by PB inspired by prevalent behavior change theory [11]. A formative research design was used in this study building on this previous work. The formative process included (1) focus groups with students and (2) panel evaluation with students and experts (see Multimedia Appendix 1). The revision process was iterative, including continuous revisions of messages and the intervention format based on data from the focus groups and panel evaluation. Changes included omitting messages, adding messages, and changing wording, content, and order of messages (see Results section). Both CL and KT revised messages and the intervention format and generated a final version. All authors read and approved the final version of the messages and intervention format. Finally, a BCT analysis was conducted to elucidate the content of the messages and their theory base.

### Focus Group Discussions

The aim of the focus groups was to explore the perspective of potential users regarding the preliminary content and structure of the messages. In addition, the first two focus groups aimed to investigate student alcohol culture and perspectives of SMS text message interventions in general. Participants were recruited through advertisements and the snowball technique at the five universities included in the study. Purposive sampling was used and included individuals from the target population. The focus group discussions lasted approximately 1.5 hours following a semistructured format and including questions on student alcohol culture, length, frequency, and timing of messages, as well as the intervention format (see Multimedia Appendix 2). Participants were shown examples of preliminary messages to gain an idea of how a message could look like. All focus group discussions were audio-recorded and transcribed. CL took part in all the focus groups. PB took part in two focus groups. On these occasions, CL and PB alternated between the roles of note taker and group facilitator.

The data from the first two focus groups were compiled by CL and summarized according to aim (ie, students’ thoughts on SMS text message support). Qualitative descriptive analysis [26] was used to analyze the data from the next five focus groups. In this analysis, the data were coded using predefined categories based on the aim of the study: length, frequency, and timing of messages and intervention format. Relevant data were identified and summarized for each category. The content of each category was expanded by revisiting the data and comparing data across categories. KT performed the qualitative descriptive data analysis.

### Panel Evaluation

The panel evaluation aimed to gain input from students and experts on specific preliminary messages. Three separate questions asked participants to rate, on a scale from 1 to 4 (1=very low degree; 4=very high degree), to what degree messages were understandable, usable, and had good tone. There was also space to write comments for each message. Thirty-three students from the five universities in Sweden that had participated in the focus groups were invited via email to complete the panel evaluation. All students were compensated 500 SEK for taking part. Twenty-one experts were invited to participate and included both researchers and staff from student health care centers. The experts were not compensated for participating.

The qualitative data were analyzed by CL using qualitative descriptive analysis [26]. The data from all participants were summarized for each message. Data on individual messages were then and potential revisions discussed. The quantitative data were analyzed by KT using descriptive statistics. Messages that were rated low or very low by at least three participants on either of the three outcomes were analyzed separately. The qualitative data for these messages were reviewed and revisions made. Each message was also categorized as information- or practice-based. Information-based messages included facts or general tips about behavior change. Behavioral practice-based messages typically asked users to reflect on or practice behavior change. The categorization of messages was performed to investigate if the results differed depending on the type of message. Both CL and KT carried out revisions of messages based on the data from the panel evaluations.

### Behavior Change Technique Analysis

The aim of the BCT analysis was to elucidate the content of the messages and their theory base. A taxonomy of BCTs to reduce excessive alcohol consumption was used in the analysis [27]. The taxonomy was developed by Michie and colleagues [22,23] and is based on previous taxonomies for behavior change and stems from behavior change theory. The techniques represent strategies, such as self-monitoring, that have been found to be effective in behavior change interventions. In this study, the BCT analysis was conducted on the final version of the intervention. Multimedia Appendix 3 includes all the SMS text messages as well as their corresponding BCT.

## Results

### Participants

#### Focus Group Discussions

Seven focus groups were conducted with students from five universities in Sweden. The participants were between 19 and 25 years of age. Of a total of 43 students, 11 were males and 32 were females. The students were attending a mixture of academic degree courses in arts and sciences.

#### Panel Evaluation

Sixteen students completed the panel evaluation (aged between 19 and 24 years; 15 females, 1 male). In total, five experts took part; two used the evaluation form and three responded as a group giving general feedback and comments to individual messages. All experts were women with a mean age of 58 (SD 12) years.

### Main Findings

#### Focus Group Discussions

Overall, students expressed positive feedback regarding the duration of the intervention and regarding receiving SMS text message support for reducing drinking. Four message characteristics that were found to be important to engage students emerged from the data: neutrality, motivating content, tangible information, and clarity. These four criteria were used to assess the messages and identify the need for revisions. Neutrality meant that the message was based on facts and, if relevant, highlighted both positive and negative aspects of alcohol:

If you keep to pure facts then I think that you take it more seriously and you take it in easier.

group 4

Exactly...and maybe not in all messages that you should try to do it that all should be a bit positive and negative but it is quite a good aspect in these other messages.

group 1

The motivating aspect included the wording or tone that aimed to increase self-esteem, self-efficacy, and motivation to make changes. It was deemed important that messages had a coaching approach instead of using scare tactics or creating feelings of anxiety or guilt:

I like the start-up messages, like, that it’s like then it’s a bit triggering or coaching like “now let’s go” for six weeks.

group 1

Yeah you should watch out for using scare tactics ’cause that can make you drop out cause it makes you feel bad cause I make bad choices.

group 4

A tangible characteristic meant that the messages were easy to read with accessible information, clear and to the point. Another aspect was a preference for contrasting examples (eg, “How much money do you spend on food versus alcohol, and do you think it is worth it?”):

I think that if you compare with food every month and it’s maybe half the amount that I spend on food what better food I could get.

group 2

...it’s also good with short and concise like you get a reminder.

group 5

Ensuring clarity of messages encompassed providing sufficient information to minimize the risk of misunderstandings and not including multiple themes within one message. The students typically expressed that they preferred short concise messages, and that messages not exceed one page or screen:

Yeah or the message is good but I think that you can reword it so that the purpose of it is clearer.

group 1

...but if there’s properly clear words, like the first ones, then you will read it.

group 3

Furthermore, the focus group participants perceived 6 weeks of messages to be an appropriate duration for the intervention. There was limited consensus among students regarding the timing and frequency of messages and their personalization. Students expressed that they read messages more thoroughly in the mornings and the evenings. During the daytime, messages could get lost or were given low priority. However, students also expressed that messages were always read, but perhaps not straight away. Moreover, students’ preferences for message frequency varied from three to four messages per week to one message every day, at least at the start of the program. On nights out, many students agreed that two messages with one message being sent late at night would be valuable. Some students thought that it would be good to have more personalized messages (eg, “Hi John”), whereas others perceived that personalized messages would be less credible.

Students described a university culture in which alcohol drinking was a social phenomenon and that heavy drinking was normalized. For example, the first weeks of term were described as an induction to heavy drinking rather than an induction to academic studies. Furthermore, students expressed that alcohol consumption was highly emphasized on university campuses through posters, flyers, special offers, and student union activities. Student unions and other student societies were believed to have a role in pushing alcohol consumption based on financial interests (ie, selling alcohol).

#### Panel Evaluation

Overall, students and experts gave high scores to the majority of the messages. The majority of messages were given a score greater than three out of a top mark of four. Messages were given a mean score of 3.86 (SD 0.43) on understanding, 3.70 (SD 0.61) on usability, and 3.78 (SD 0.53) regarding tone. Messages were rated high irrespective of the type of message. Messages with primarily information content were given a mean score of 3.84 (SD 0.43) on understanding, 3.72 (SD 0.60) on usability, and 3.78 (SD 0.56) on tone. Similarly, practice-based messages were given a mean score of 3.85 (SD 0.44) on understanding, 3.71 (SD 0.60), on usability, and 3.78 (SD 0.54) on the tone of the message. Students and experts scored messages similarly with a mean score greater than three for the majority of messages regarding all three outcome measures: understanding, relevance, and tone. Thirteen of the 70 messages were rated low or very low on either understanding, usability, or tone by at least three participants (experts or participants). These messages were analyzed separately; the qualitative data were consulted and subsequent revisions made. The distribution of low scores was equal for understanding, usability, and tone.

Qualitative feedback was given for 67 of 70 messages and included suggestions for change in wording, change in the order of messages, and feedback on why a message was unclear or needed major revision. For example, a message that included tips on how to prepare for saying no to a drink on the next night out received feedback that it was too long and that it had a confusing message. The message was shortened and the wording made more concise. The data also included positive feedback that confirmed the value of messages. For example, messages that were to the point and included tangible examples (eg, “How much money do you spend on alcohol in a typical week compared with food?”).

### Behavior Change Technique Analysis

Twenty-three techniques were identified in the final version of the intervention (62 messages). Some techniques were used in more than one message or across two messages. The techniques aimed to motivate students to reduce their alcohol consumption, address self-regulation, increase self-efficacy, and increase students’ awareness of social and professional support. Table 1 shows the BCTs for each message and their function presented per week.

**Table 1:** Behavior change technique and function for each message per week.

Message by week^a^		Behavior change technique	Function
**Week 1**			
	1-2	Provide feedback on performance in relation to behavior goal*	Motivation
	3	Provide tips on how to perform the behavior	Motivation
	4-5	Provide information on consequences of excessive drinking	Motivation
	6	Identify reasons for wanting and not wanting to reduce drinking	Motivation
	7	Assess current and past drinking behavior	Motivation
	8	Provide tips on how to perform the behavior	Motivation
	9	Prompt to practice refusal	Self-efficacy
	10	Advise on use of social support	Social influence
	11	Provide information on the consequences excessive drinking	Motivation
**Week 2**			
	12	Provide instruction on how to perform the behavior	Self-efficacy
	13-14	Provide feedback on performance in relation to behavior goal^b^	Motivation
	15	Provide information on the consequences of excessive alcohol consumption	Motivation
	16	Identify reasons for (not) wanting to reduce excessive alcohol consumption	Motivation
	17	Assess past history of attempts to reduce excessive alcohol consumption	Motivation
	18	Provide information on the consequences of excessive alcohol consumption	Motivation
	19	Advise on environmental restructuring	Self-regulation
	20	Prompt reflection on approval of others	Social influence
	21	Provide information on the consequences of excessive alcohol	Motivation
	22	Prompt reflection on approval of others	Motivation
**Week 3**			
	23	Prompt reflection on approval of others	Motivation
	24-25	Provide feedback on performance in relation to behavior goal^b^	Motivation
	26	Provide information on the consequences of excessive alcohol	Motivation
	27	Behavior substitution	Motivation
	28	Prompt practice	Self-regulation
	29	Provide information on the consequences of excessive alcohol	Motivation
	30	Identify reasons for (not) wanting to reduce excessive alcohol consumption	Motivation
	31	Provide information on the consequences of excessive alcohol	Motivation
	32	Facilitate identification of barriers and problem solving	Self-regulation
	33	Provide instruction on how to perform the behavior	Self-efficacy
**Week 4**			
	34-35	Provide feedback on performance in relation to behavior goal^b^	Motivation
	36-37	Prompt review of goals	Self-regulation
	38	Behavior substitution	Self-regulation
	39	Provide information on the consequences of excessive alcohol	Motivation
	40	Facilitate identification of barriers and problem solving	Self-regulation
	41-43	Provide instruction on how to perform the behavior	Self-efficacy
	44	Provide information on the consequences of excessive alcohol consumption	Motivation
			
			
**Week 5**			
	45-46	Provide feedback on performance in relation to behavior goal^b^	Motivation
	47	Assess current and past drinking behavior	Self-regulation
	48	Advise on avoidance of social cues for drinking	Self-regulation
	49	Provide information on the consequences of excessive alcohol consumption	Motivation
	50	Identify reasons for (not) wanting to reduce excessive alcohol consumption	Motivation
	51	Give options for additional and later support	Social influence
	52	Prompt self-recording	Self-regulation
	53	Identify reasons for (not) wanting to reduce excessive alcohol consumption	Motivation
**Week 6**			
	54	Prompt self-recording	Self-regulation
	55-56	Provide feedback on performance in relation to behavior goal^b^	Motivation
	57	Emphasize choice	Self-efficacy
	58	Environmental restructuring	Self-regulation
	59	Prompt practice	Self-efficacy
	60	Anticipated regret	Motivation
	61-62	Provide feedback on performance in relation to behavior goal^b^	Motivation

^a^Each week started on a Sunday.

^b^Paired messages that were repeated each week.

### The Final Program Design

Significant changes were made to the messages in the formative process. The final intervention included a 6-week program with a total of 62 messages (see Multimedia Appendix 3). The initial preliminary intervention consisted of 57 unique messages. Thus, changes included omitting and adding messages, changing the wording and order of messages, and splitting and combining messages. All the original 57 messages were altered at least once.

The final version of the intervention commenced with students receiving an email asking them to set a goal of how much they would like to reduce their drinking. They could then register for the intervention by sending their mobile number to receive the first message. Participants could unsubscribe to the messages at any time by responding the word “STOP” to any of the SMS text messages.

The first 4 weeks of the program had a higher frequency of messages, nine each week, followed by seven messages in weeks 2 to 5 and five messages in week 6. Messages were sent 7 days a week at various times around midday, late afternoon, or early evening. Forty-eight of the 62 messages were unique. Two messages were repeated at the start of each week; students were asked to report the number of drinks they had the previous week via SMS text message. Subsequently, they received a second SMS text message including feedback on their performance in relation to their goal set at the start of the intervention. These “paired” messages were repeated every Sunday. The content of the unique messages were primarily information-based or behavioral practice-based. Information-based messages typically included facts about alcohol and health, the consequences of excessive drinking, or tips about behavior change strategies. Behavioral practice-based messages included asking students to reflect on or practice behavior change; for example, reflect on triggers for excessive drinking or practicing saying no to drinking on a night out.

The SMS text messages will be sent from GSM modems, programmed and operated by one of the authors (MB). The technical platform has been used in previous studies and proven to work without any major interruptions.

## Discussion

### Principal Findings

We set out to develop an SMS text message intervention targeting excessive drinking among university students using formative research. The formative process resulted in significant changes to the original messages. The findings showed that neutral, motivated, clear, and tangible messages engaged students. However, there was limited consensus among students regarding optimal timing, frequency, and personalization of messages, although the students agreed that a 6-week duration of the program was acceptable. The panel evaluation showed overall positive scores on the understanding, usability, and tone for the majority of the messages. The panel also received comments for most of the messages. The BCT analysis identified 23 techniques used in the final intervention. The final program design resulted in a 6-week intervention including 62 messages sent with varied frequency and timing.

### Comparison With Previous Work

In line with previous research, these findings show that text messaging has the potential to be a useful tool for interventions targeting excessive drinking among students [15]. Overall, the findings showed that students were positive toward receiving support through SMS text messages. However, there was limited consensus among students regarding the timing, frequency, and personalization of messages. To create optimal timing schedules for specific target groups could prove to be a challenge for SMS text message interventions. Previous studies have attempted to solve this by allowing users to decide on the content and timing of messages via a website [28]. However, more research is needed on the effectiveness of user-created compared with preset schedules. Furthermore, our findings showed that messages were read, if not straightaway, at least the same day, suggesting that the exact timing of a message may be less relevant. This could be especially true for messages that aim to evoke reflection (that can be done at any time) compared with reminders or prompts, which would be more valuable at certain time points (eg, a reminder to drink water on a night out).

Engaging users is a central aspect of optimizing the effectiveness of SMS text message interventions. A recent study showed that SMS text messages that aimed to reduce hazardous drinking were only perceived to be moderately helpful and interesting among a population of young adults. The study highlighted the challenge of engaging this target group in alcohol prevention [29]. Our findings showed that messages that were neutral, motivated, clear, and tangible were more engaging and likeable. These findings support previous research that messages need to be supportive and have a positive tone. Bock et al [15] found that students appreciated making wise choices and did not want to be told not to drink. Techniques used in face-to-face interventions could be applied in SMS text message interventions [30]. Techniques relating to building rapport and general support can be more difficult to achieve via text messaging due to their interactive components. The findings from this study, in support of previous research, showed that the tone of messages is important to engage users. Other engagement strategies that have been suggested are ease of use, user ability to change the design, and tailored information [15,30]. Furthermore, a study that asked users to choose one of two messages with slightly different wording showed the importance of the wording of messages to engage users. Similar to our findings, users preferred messages that highlighted the positive over the negative, were nonaggressive, and benefit-oriented messages. In addition, aspects such as correct spelling and grammar, directive rather than passive approach, and messages without “textese” (eg, abbreviations) were found to be more preferred [31].

The notion of tailored interventions can be derived from the elaboration likelihood model (ELM). ELM proposes that information that is perceived to be personally relevant and expressed by a reliable sender increases perceived acceptability [32], which has also found empirical support [33]. A review on the main aspects of computer-tailored health interventions showed that tailoring was typically achieved through giving tailored feedback, personalization, and matching content with different groups of users [34]. However, tailoring has also been found to be complex and influenced by, for example, target groups and demographic variables [35]. Similarly, our findings showed limited consensus among students regarding tailoring, for example, personalization of messages. In order to optimize tailoring, intervention developers must have a sound knowledge of the culture, needs, and preferences of the end users. Although formative research designs would be valuable in this process, more research is needed on the opportunities with SMS text message interventions regarding tailoring. That is, what are the technical opportunities and challenges with tailoring and what level of tailoring is optimal, such as individual user-created messages versus group tailored information.

Our BCT analysis identified 23 techniques in the final program. In a Delphie study, international experts reached consensus on four BCTs that were estimated to be effective for mobile interventions targeting alcohol consumption. The techniques that received the highest mean ranking were for example goal setting, self-monitoring, action planning, and feedback in relation to goals [30]. Most of these techniques were used in our intervention and derived from, for example, control theory [36] and goal-setting theory [37]. Furthermore, these BCTs aim to tap into varied aspects of the self-regulatory process whereby the individual continuously monitors and adjusts discrepancies between an ideal or goal and actual states of being [38]. There is empirical support for interventions that address self-regulatory skills. A Cochrane review showed that receiving normative feedback had a small effect on reducing alcohol consumption in student populations [39]. Furthermore, self-monitoring has been found to be associated with improved effectiveness of brief interventions [27]. An earlier study found that reporting alcohol use on a daily basis reduced drinking among heavy drinkers by 20% [40]. There is also empirical support for including “if...then” tasks in interventions (eg, action planning) for a variety of behaviors [41] and student populations [42]. Finally, in a review on effective interventions to reduce risky drinking, goal setting, feedback, and advice were found to be the most effective strategies [43]. Thus, addressing self-regulatory skills in SMS text message interventions is feasible and, considering its empirical support, important in future intervention development.

In addition to self-regulatory skills, our intervention included BCTs that address motivation, such as anticipated regret. Looking at prevalent behavior change theory (eg, social cognition models [44,45] and social cognitive theory [46]), motivation to change is a central tenet. These theories argue that behavior change is a function of modified attitudes and beliefs about a behavior and perceptions about one’s ability to make changes. Other BCTs that derive from these theories, and that were included in our intervention, give information on the consequences of drinking or prompt reflection on the reasons to reduce drinking [27].

### Strengths and Limitations

A strength of this study could be the formative study design, which entailed revision of all messages based on both user and expert feedback. We believe that the input from the target audience and experts enhanced the intervention. A future randomized controlled trial will investigate the effect of the intervention on alcohol consumption among students. Another strength of the study is the use of BCT analysis, which elucidated the theory base of the messages, giving readers a better understanding of what the intervention entails and enabling comparison with other interventions. A limitation of the study could be that most of the student participants in both the focus groups and panel evaluation were women. However, men were included in the data and messages were revised to interest both sexes. Another limitation could be that some participants took part in both the focus groups and the panel survey (10 of 21). The fact that individuals participated in focus groups prior completing the panel survey may have introduced bias. However, participating in both focus groups and the panel survey could also have been an advantage because these participants had a thorough understanding of the program. Lastly, the BCT analysis was performed by only one researcher, which may have limited the analysis compared with using several raters.

### Implications

The study followed the recommended steps for the development of SMS text message-based interventions [19]. The findings show the importance and value of using a formative process in intervention development. The content of the SMS text messages was significantly revised based on input from end users and experts. The findings showed that neutral, motivated, clear, and tangible messages engaged students. These findings could be used in future studies aiming to develop SMS text message-based interventions. Furthermore, drinking patterns and access to mobile phones among university students in Sweden are similar to those in other Western countries. Therefore, we believe that the findings can be generalized to other university contexts.

### Conclusions

The formative research design was valuable and resulted in significant changes in the intervention. All the original SMS text messages were changed and new messages were added. Overall, the findings showed that students were positive toward receiving support through SMS text messages and that neutral, motivated, clear, and tangible messages promoted engagement. More research is needed on the timing, frequency, and tailoring of messages.

## Appendix

Multimedia Appendix 1 The formative research design.

Multimedia Appendix 2 Focus group interview guide.

Multimedia Appendix 3 List of SMS messages in the final program design.

## Abbreviations

BCT: behavior change technique
ELM: elaboration likelihood model
SMS: short message service
