# Supplementary material for: Text Message-Based Intervention Targeting Alcohol Consumption Among University Students: Findings From a Formative Development Study
Source: JMIR Mhealth Uhealth. 2016 Oct 20;4(4):e119. doi: 10.2196/mhealth.5863 (PMC5095367; doi:10.2196/mhealth.5863)

Multimedia Appendix 1

Multimedia Appendix 1: Figure of the formative research design used in the study including brief information on participants.

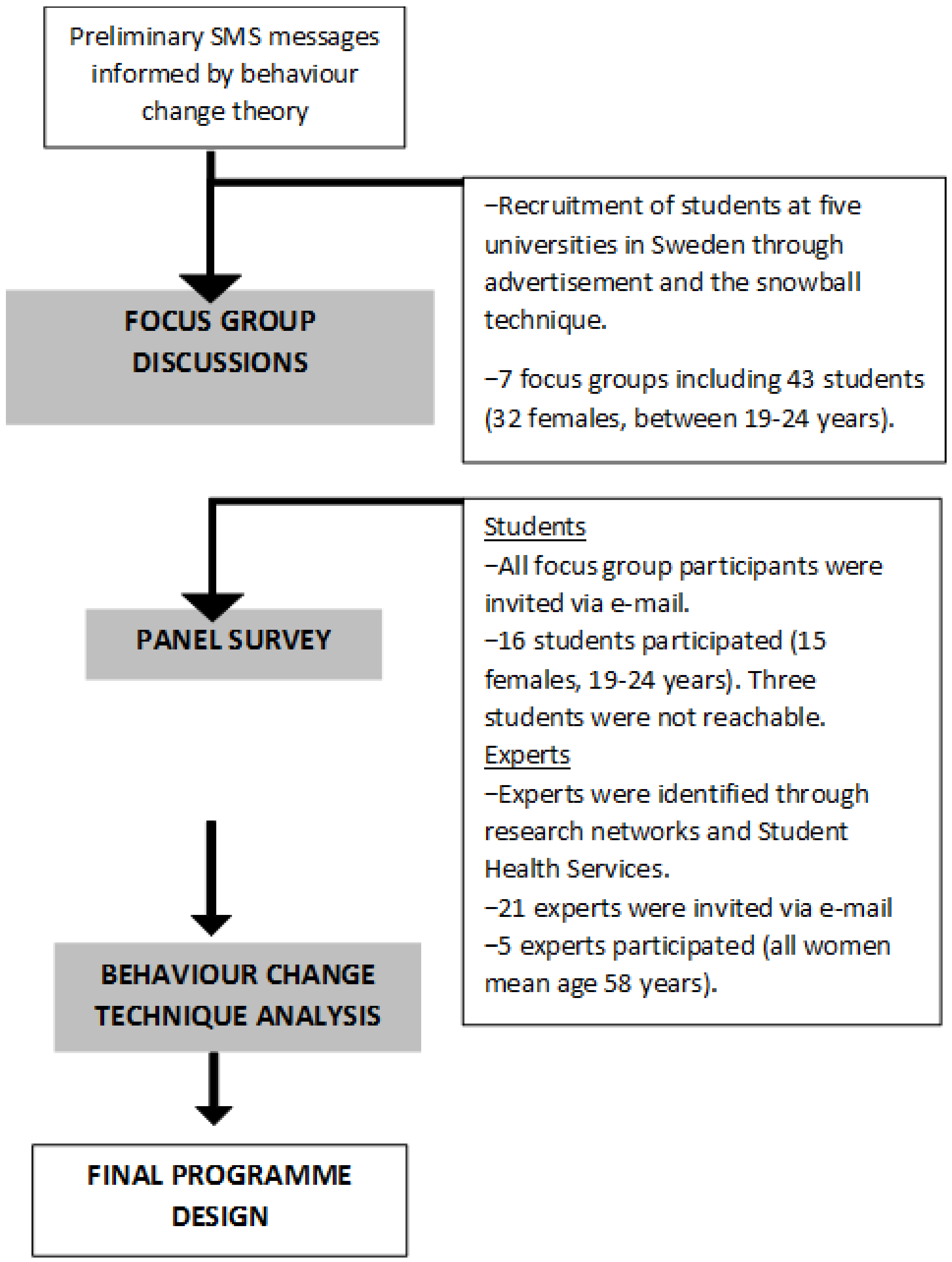

Supplement: Multimedia Appendix 1 [file mhealth_v4i4e119_app1.pdf]
