# Supplementary material for: Text Message-Based Intervention Targeting Alcohol Consumption Among University Students: Findings From a Formative Development Study
Source: JMIR Mhealth Uhealth. 2016 Oct 20;4(4):e119. doi: 10.2196/mhealth.5863 (PMC5095367; doi:10.2196/mhealth.5863)
Supplement: Multimedia Appendix 2 [file mhealth_v4i4e119_app2.pdf]

Multimedia Appendix 2

Interview guide used during the focus groups:

QUESTIONS	SUB-QUESTIONS/PROMPTS[1]
**Alcohol culture**	
1. Why do you think students drink?	Parties, stress, fun etc?<br>How does a typical week look like?<br>How do you drink compare to other students do you think?
2. If you would like to reduce your drinking, what support would be useful?	
3. What support do you think others need to reduce their drinking?	
**Intervention format**	
Do you have any thoughts on how a SMS-based service should look like?	Is 6-8 week long service realistic, do you have any other ideas of format?
What do you think about the amount and frequency of messages that are optimal?	Optimal days?<br>Optimal time?<br>When is messages read?<br>Optimal number of messages per day?
**SMS messages**	
We are planning to include messages containing factual information, practical exercises, challenges and those that prompt reflection. What are your thoughts about this?	
What are your thoughts about the length of messages?	
What are your thoughts about the wording and content of the messages?	
Are there any type of message that you would add to the programme?	
We are considering taking a personal approach within the messages and greet the user by name. What are your thoughts about that?	What are the pros and cons?
[1]Used if needed.	
